# Evaluating the Dimensionality and Psychometric Properties of the Brief Self-Control Scale Amongst Chinese University Students

**DOI:** 10.3389/fpsyg.2019.02903

**Published:** 2020-01-08

**Authors:** Sai-fu Fung, Chris Yiu Wah Kong, Qian Huang

**Affiliations:** ^1^Department of Social and Behavioural Sciences, City University of Hong Kong, Kowloon, Hong Kong; ^2^Department of Sports Training, Xi’an Physical Education University, Xi’an, China

**Keywords:** Brief Self-Control Scale, Chinese, confirmatory factor analysis, personality, self-control, university students, validation

## Abstract

The aim of this study was to assess the dimensionality and psychometric properties of the Brief Self-Control Scale (BSCS) using a sample of university students in mainland China. Nine hundred and three students from a Chinese university participated in this study. The internal consistency, criterion validity, factorial validity and construct validity of the scale were examined. The Chinese versions of the BSCS demonstrated good internal consistency with a Cronbach’s alpha of 0.81. The BSCS also showed significant moderate correlations with other construct-related scales. Exploratory factor analysis (EFA) and confirmatory factor analysis (CFA) suggested that only a modified 11-item BSCS with a four-factor structure was a good model fit in the sample of Chinese university students, as χ^2^ (106.626)/37 = 2.88, SRMR = 0.036, comparative fit index (CFI) = 0.992, Tucker-Lewis fit index (TLI) = 0.989, RMSEA = 0.046. The implications for research and theoretical development are discussed.

## Introduction

Since the inception of impulse control and self-control concepts in the early 70s there has been extensive empirical research on their psychometric properties, theoretical underpinnings, and behavioral implications (Mischel, 1974; Ainslie, 1975). Many scholars regard self-control as essential for human positive growth and development (Metcalfe and Mischel, 1999; Tangney et al., 2004; Duckworth and Kern, 2011; de Ridder et al., 2012). Twentieth-century measurements of self-control, such as the self-control rating scale (Kendall and Wilcox, 1979), the bonding self-control scale (SCS) (Gottfredson, 1990), and Grasmick’s SCS (Grasmick et al., 1993), were commonly used for criminological and addictive studies amongst children and juvenile delinquents. These scales were evaluated and applied to different criminological research projects involving children and juveniles (Wang, 2002; Piquero and Bouffard, 2007; Weng and Chui, 2018). Studies suggest that whilst people with higher self-control are inclined to delay gratification and are high achievers, those with lower self-control are less likely to inhibit impulsive behavior (Mischel and Mischel, 1983; Baumeister, 2016). SCSs have been used to analyse the relationship between emotional exhaustion and counterproductive workplace behaviors. In particular, Maloney et al. (2012) found that impulsivity was positively and significantly related to both interpersonally directed and organizationally directed counterproductive workplace behaviors, whereas restraint was negatively related to emotional exhaustion when controlling for the effects of impulsivity. Research also suggests that self-control is an important risk and protective factor amongst jail inmates (Malouf et al., 2014).

In the literature on personality, self-control has been recently associated with positive psychological adjustment and a broad range of positive outcomes in life, such as happiness, well-being and quality of life (Rothbaum et al., 1982; Tangney, 1991, 1995; Baumeister, 1994; Tangney et al., 1996; Eisenberg et al., 1998; Fabes et al., 1999). As such, Tangney et al. (2004) had developed the 36-item SCS and the shortened 13-item Brief Self-Control Scale (BSCS). The development and validation of these two scales signifies that self-control concepts can be more scientifically applied to various types of performance such as academic attainment, the formation of good habits, refraining from distractions and controlling of urges and impulsive behavior such as procrastination and drug-taking.

Brief Self-Control Scale has been translated into different languages and validated by the French-speaking population of Canada (Brevers et al., 2017), and in Germany (Bertrams and Dickhäuser, 2009) and Turkey (Nebioglu et al., 2012). However, the validation and application of the full and BSCS scales in China is still in its infancy. An initial study conducted in Chinese amongst college students in Wuhan suggested that the full version of SCS supports a five-factor construct scale (Tan and Guo, 2008), which was then used to examine the patterns of mobile phone usage amongst the students (Jiang and Zhao, 2016). Unger et al. (2016) proposed validating Tangney and associates’ SCS in mainland China, and attempted to investigate the psychometric properties of SCS and BSCS using 371 Chinese college students between 17 and 23 years old. They found that both scales had a satisfactory internal consistency and a reasonable goodness of fit for the five-factor construct. They concluded that the BSCS was preferable to the SCS as it had a strong correlation with the full scale but saved time and had a higher rate of return.

The aim of this study is to re-examine the 13-item BSCS in two ways. First, it evaluates the issue of dimensionality of the BSCS. The literature continues to be controversial with regard to the multi-factor structure of the BSCS. The original scale developers and the subsequent validation studies replicated the five-factor structure, i.e., general capacity for self-discipline (5 items), inclination toward deliberate or non-impulsive action (3 items), healthy habits (2 items), self-regulation in service to build a strong work ethic (2 items), and reliability (1 item) (Tangney et al., 2004; Unger et al., 2016). Since the introduction of SCSs in 2004, scholars have offered other conceptualizations of self-control with different dimensions (Fulford et al., 2008; Friese and Hofmann, 2009) and have proposed different conceptualizations of two-factor structures on the basis of the existing 13-item BSCS, such as general self-discipline (9 items) and impulse control (4 items) (Ferrari et al., 2009). Maloney et al. (2012) proposed an 8-item BSCS, focusing on impulsivity (4 items) and restrain (4 item). Alternatively, a 10-item BSCS, emphasizing inhibition (6 items) and initiation (4 items) was suggested by de Ridder et al. (2011). Lindner et al. (2015) attempted to evaluate the above two-dimensional BSCS specifications, but could not demonstrate which conceptualization of the BSCS was more appealing. Hence, evaluating the dimensionality of the Chinese version of BSCS warrants attention.

Second, the Chinese version of BSCS’s psychometric properties is subject to further investigation. Unger et al. (2016) have attempted to validate the Chinese version of BSCS in China, however, their study with potential limitations like small sample size and inadequate evaluation of criterion validity. Hence, the design of this study in particular, pays closer attention to the criterion validity of self-control with other construct-related scales related to the conceptualization of self-control. Furthermore, some low factors loadings of the scale items needed retesting to confirm whether they need replaced.

## Materials and Methods

### Participants

This cross-sectional study recruited 903 respondents from Huashang College, Guangdong University of Business Studies, located in the southern part of China. The gender ratio of the sample (792 females to 111 males) matched that of the official school record, i.e., over 80% of the students enrolled in the university were female. The average age of the respondents was 20.56 years (SD = 2.753). Student sample profiles of this study matched those of the original scale developers who had recruited 28% male and 72% female and 19% male and 81% female university students in study 1 and study 2 samples, respectively (Tangney et al., 2004).

### Measures

The full version of the SCS comprises of 36 items. The original scale developers proposed using the shortened version, the BSCS, which contains 13 items, including 1, 2, 3, 4, 6, 13, 17, 22, 28, 29, 30, 31, and 32. These 13 items were rated on a 5-point Likert scale ranging from 1, *not at all like me*, to 5, *very much like me*. Eight items, including 2, 3, 4, 6, 17, 28, 29, and 31 had reversed scores (Tangney et al., 2004). The reversed items were re-coded in the dataset prior to the analysis.

The Chinese version of the BSCS was adapted from Unger et al. (2016). We recruited two translators who were fluent in both English and Chinese to cross-check the translated versions to verify whether the original English and Chinese versions were identical (Brislin, 1970). To further ensure that the translated versions were free from any cultural biases, two pilot studies were conducted in Xi’an and Guangzhou, located in northern China and southern China, respectively. Each pilot study involved five mainland Chinese university students from diverse academic backgrounds, ranging from accountancy and management to sports sciences, computer sciences, and the social sciences. None of the participants reported any difficulties in understanding and answering the questions. Data from the pilot studies were excluded in the dataset.

### Procedures

The research team used the announcement function in the school-based intranet smartphone application available in both iOS and Android operating systems to recruit students voluntarily participate in an online self-reported survey related to self-control, well-being and Internet usage from June to July 2018. On the questionnaire page, students were fully informed the background of the study and we obtained informed consent from the participants prior to allow them to complete the self-administered questionnaire. The respondents were only able to submit the completed questionnaire once. Each participant spent around 10 min completing the questionnaire. The data that we collected were anonymous. The study was approved by the ethical committee of the Huashang College, Guangdong University of Business Studies. The entire research process and data collection procedure also complied with the ethical standards of the Declaration of Helsinki and the relevant government policies stipulated in the Article 14 of Chapter III, Statistics Law of the People’s Republic of China.

Various psychometric testing tools and validated instruments were used to examine the BSCS. The internal consistency of the BSCS was assessed by Cronbach’s alpha (Cronbach, 1951), McDonald’s Omega (McDonald, 1999; Zinbarg et al., 2005; Revelle and Zinbarg, 2009) and the corrected item-total correlations between all the 13 items were examined (Hair, 2010; Tabachnick, 2013). The criterion validity was evaluated with other validation constructs or measurements reported in relevant studies on self-control as well as the item-to-scale correlations (Beaton et al., 2000; Loewenthal, 2001). According to Tangney et al. (2004) and Unger et al. (2016), the SCS is positively correlated with self-esteem, happiness, quality of life, and well-being, but has significant moderate negative correlations with psychometric instruments related to psychological problems and symptoms of psychopathology, such as the 12-item General Health Questionnaire (GHQ-12). Owing to the availability of the validated Chinese scales and the length of the questionnaires, five well-established instruments were used to evaluate the criterion validity of the BSCS: The GHQ-12 evaluated by twelve items (with five reversed items) to assess the severity of health related problems using a 4-point Likert-type scale. Respondents with high scores indicate worse health (Goldberg and Williams, 1991); Rosenberg Self-esteem Scale (RSES) consists of ten statements (with five reversed items) evaluated by 4-point Likert-type scale, with 1 = *strongly disagree* and 4 = *strongly agree*. High scores refer to high level of self-esteem (Rosenberg, 1965; Rosenberg et al., 1989); Satisfaction with Life Scale (SWLS) comprised of five items with 7-point Likert-type scale (1 = *strongly disagree*; 7 = *strongly agree*). High scores signify the respondents highly satisfied with their life (Diener et al., 1985; Pavot et al., 1991; Pavot and Diener, 1993, 2008); Subjective Happiness Scale (SHS) consists of four statements measured by 7-point Likert-type scale. High scores mean happier (Lyubomirsky and Lepper, 1999); and WHO (Five) Well-Being Index (WHO-5) comprised of five items with 6-point Likert-type scale (0 = *at no time*; 5 = *all of the time*), high score indicates high level of well-being (Bech et al., 2003; Bech, 2004, 2012). In addition to the original 13-item BSCS (Tangney et al., 2004) and several basic demographic questions, the participants were asked to complete a questionnaire with 51 items.

The evaluation of the scale’s factorial validity was based on exploratory factor analysis (EFA). There are controversies about the rotation method used in the EFA (Jennrich and Sampson, 1966). Current BSCS studies use different EFA extraction methods, thereby giving rise to controversies with regard to the multi-factor structure. For example, a recent study used principal components with direct oblimin rotation (Maloney et al., 2012); Ferrari et al. (2009) used the maximum likelihood process with varimax rotation. However, the original scale developers used principal components with varimax to trim the SCS scale from 36 to 13 items (Tangney et al., 2004). The varimax is a commonly used orthogonal factor rotation method for simplified factor structures (Hair, 2010). Hence, we adopted principal components with varimax as an EFA rotation method, which is the same as the originally developed scale, to evaluate the Chinese version of the BSCS. Due to a relatively large sample size, i.e., over 350 respondents in this study; hence, an item with a factor loading over 0.50 can be interpreted as having practical significance (Hair, 2010).

Confirmatory factor analysis (CFA) was used to examine the construct validity of the scale (Jöreskog, 1969; Loewenthal, 2001; Brown, 2014). Although it has been argued that the maximum likelihood estimator is inappropriate for the ordinal nature of the BSCS (Lionetti et al., 2016), existing studies have predominantly used it in CFA (de Ridder et al., 2011; Maloney et al., 2012; Lindner et al., 2015; Unger et al., 2016). To address this issue, CFA has been conducted to examine the factor structure of the BSCS using the diagonally weighted least squares (DWLS) method. The usage of the DWLS estimator, which is suitable for ordinal items constructed scales, and is an effective tool for evaluating the dimensionality and psychometric properties of BSCS in the following two reasons. The BSCS as a latent construct is estimated by Likert scale items consisting of ordinal data, and the DWLS method is regarded as having a less biased and more optimal fit (DiStefano and Morgan, 2014; Li, 2016; Lionetti et al., 2016). In addition, the results of this study can be directly compared with other BSCS validation studies using frequentist estimations (de Ridder et al., 2011; Maloney et al., 2012; Nebioglu et al., 2012; Lindner et al., 2015; Unger et al., 2016). The model fit and cut-off criteria were evaluated on the basis of the following cut-off values; a comparative fit index (CFI) and a Tucker-Lewis fit index (TLI) of over 0.950, a standardized root mean square residual (SRMR) under 0.08 and an root mean square error of approximation (RMSEA) under 0.06, which were considered good fits (Browne and Cudeck, 1993; Hu and Bentler, 1999; Schreiber et al., 2006; Hair, 2010; Bass et al., 2016). An acceptable model can also be indicated by χ^2/^df ≤ 3 due to the large sample size (Bentler and Bonett, 1980; Kline, 2005). The analyses were implemented with the IBM SPSS 25.0 and the lavaan package version 0.6-3 (Rosseel, 2012) in R version 3.5.2.

## Results

### Internal Consistency

Table 1 shows the means, standard deviations, skewness, kurtosis, corrected item-total correlations, and Cronbach’s alpha if items were deleted of the BSCS (*N* = 903). The mean score for the BSCS among all the respondents, male and female were 38.77 (SD = 7.32), 39.33 (SD = 7.35), and 38.68 (SD = 7.32), respectively, which is similar to that reported in the original study (Tangney et al., 2004). No significant differences and relationship were observed in the scale scores on sex of the respondent based on the independent-sample *t*-test and correlation results. The corrected item-to-total correlations in the 13-item BSCS ranged from 0.077 to 0.550. The following two items reported values lower than 0.300: BSCS17 (0.077) and BSCS13 (0.196). This finding was addressed in the subsequent EFA while evaluating the scale’s factorial validity. The Cronbach’s alpha of the BSCS in this study was 0.80, replicating the original BSCS Cronbach’s alpha values, i.e., 0.83 and 0.85 in studies 1 and 2, respectively (Tangney et al., 2004). The results suggested that the scale is highly reliable in terms of internal consistency.

**TABLE 1:** Descriptive statistics for 13-item BSCS scale.

**Item**	**Mean**	***SD***	**Skewness**	**Kurtosis**	**Corrected item-total correlation**	**Cronbach’s alpha if items were deleted**
BSCS1	3.38	0.970	–0.271	–0.077	0.440	0.782
BSCS2	2.79	1.018	0.044	–0.408	0.534	0.773
BSCS3	2.58	1.089	0.250	–0.454	0.550	0.771
BSCS4	3.20	1.063	–0.161	–0.488	0.473	0.779
BSCS6	3.67	1.150	–0.501	–0.614	0.459	0.780
BSCS13	3.78	1.052	–0.601	–0.260	0.196	0.803
BSCS17	1.82	0.873	0.830	0.176	0.077	0.808
BSCS22	3.04	1.058	–0.076	–0.425	0.432	0.782
BSCS28	2.44	1.060	0.402	–0.379	0.386	0.786
BSCS29	2.87	1.038	0.018	–0.375	0.552	0.772
BSCS30	3.20	0.982	–0.091	–0.205	0.398	0.785
BSCS31	2.82	1.101	0.169	–0.590	0.515	0.774
BSCS32	3.19	1.113	–0.059	–0.648	0.500	0.776

### Criterion Validity

According to Tangney et al. (2004), self-control is one of the most powerful and beneficial aspects of the human psyche, and is positively related to happiness and health. The BSCS is demonstrated to have significant moderate positive correlations with self-esteem, quality of life and well-being (Tangney et al., 2004; Unger et al., 2016). As shown in Table 2, the Chinese version of the BSCS also showed significant moderate correlations with RSES (*r* = 0.459, *p* < 0.001), SWLS (*r* = 0.302, *p* < 0.001), SHS (*r* = 0.332, *p* < 0.001), and WHO-5 (*r* = 0.243, *p* < 0.001).

**TABLE 2:** Correlation between 13-item BSCS scale in relation to other validation constructs.

**Other construct-related scales**	**BSCS**
Rosenberg Self-esteem Scale (RSES)	0.459***
Satisfaction with Life Scale (SWLS)	0.302***
Subjective Happiness Scale (SHS)	0.332***
WHO (Five) Well-Being Index (WHO-5)	0.243***
12-item General Health Questionnaire (GHQ-12)	–0.422***

***p < 0.001.

To further evaluate the criterion validity of the BSCS, whether the scale demonstrated a negative relationship with the psychological symptoms-related scale was also assessed. The results of the correlation show that the Chinese version of the BSCS demonstrated a significant moderate negative relationship with GHQ-12 (*r* = −0.422, *p* < 0.001). This finding also replicated the existing studies’ findings in terms of the direction and magnitude of the scales related to mental disorder (Tangney et al., 2004; Unger et al., 2016). Table 3 shows the correlations between specific items and other construct-related scales. However, BSCS17 in particular, showed a very weak association with other scales, suggesting an opposite correlation orientation in the RSES, SHS, and GHQ-12 scales. In short, the 13-item BSCS demonstrated good criterion validity with the other validation constructs.

**TABLE 3:** Correlations between the BSCS items and other construct-related scales.

**Item**	**BSCS**	**RSES**	**SWLS**	**SHS**	**WHO-5**	**GHQ-12**
BSCS1	0.543***	0.285***	0.301***	0.247***	0.274***	–0.302***
BSCS2	0.629***	0.258***	0.129***	0.145***	0.124***	–0.210***
BSCS3	0.649***	0.284**	0.216***	0.192***	0.154***	–0.232***
BSCS4	0.582***	0.328***	0.114***	0.231***	0.060	–0.279***
BSCS6	0.579***	0.260***	0.028	0.145***	0.033	–0.267***
BSCS13	0.332***	0.192***	0.113***	0.162***	0.065*	–0.200***
BSCS17	0.195***	−0.085*	–0.009	−0.075*	0.021	0.086**
BSCS22	0.546***	0.230***	0.274***	0.192***	0.219***	–0.210***
BSCS28	0.506***	0.176***	0.100**	0.125***	0.106**	–0.179***
BSCS29	0.646***	0.347***	0.243***	0.247***	0.185***	–0.342***
BSCS30	0.508***	0.310***	0.332***	0.230***	0.244***	–0.273***
BSCS31	0.622***	0.264***	0.172***	0.197***	0.141***	–0.217***
BSCS32	0.610***	0.318***	0.111***	0.252***	0.091**	–0.282***

*p < 0.05, **p < 0.01, ***p < 0.001.

### Factorial Validity

Table 4 shows the results of the EFA using principal component analysis with varimax rotation as adopted by the original scale developers, who extracted five factors from the scale (Tangney et al., 2004). The explanation power of the factors relative to the total variance is explained as follows: Factor 1 explaining 17.9% of the variance consists of five items, including BSCS4, BSCS6, BSCS13, BSCS31, and BSCS32, related to the general capacity for self-discipline. BSCS13 has a factor loading of 0.470 only, which is slightly lower than the practical and significant value of 0.500; Factor 2, which is related to inclination toward deliberate/non-impulsive action consists of items BSCS1, BSCS22, and BSCS30, yielding 15.6% explanation power; Factor 3 explaining 12.3% of the variance, which is related to healthy habits consists of BSCS2 and BSCS3; Factor 4, which is related to self-regulation in service for building a strong work ethic consists of items BSCS28 and BSCS29, with 11.7% explanation power; and Factor 5 is related to reliability with item BSCS17 explaining 9.0% of the variance. The above results are identical to those of the five-factor model suggested in the original study (Tangney et al., 2004). By removing BSCS13 and BSCS17 from the scale, the EFA results of the 11-item BSCS with a four-factor structure suggested that all of the factor loadings in each factor ranged from 0.594 to 0.974 and that it supported a scale construction. The EFA results showed that the assertion of a two-factor structure suggested in the BSCS literature (Ferrari et al., 2009; de Ridder et al., 2011; Maloney et al., 2012) is not supported in this study.

**TABLE 4:** Factor loading for the Brief Self-Control Scale.

	**13-item BSCS with 5-factor structure**					**11-item BSCS with 4-factor structure**			
**Item**	**F1**	**F2**	**F3**	**F4**	**F5**	**F1**	**F2**	**F3**	**F4**
BSCS1	0.261	0.754	–0.050	0.091	0.117	0.278	0.752	–0.062	0.104
BSCS2	0.242	0.169	0.733	0.196	0.008	0.197	0.175	0.757	0.199
BSCS3	0.222	0.262	0.726	0.156	0.137	0.176	0.271	0.731	0.200
BSCS4	0.644	–0.023	0.399	0.039	0.080	0.594	–0.044	0.466	0.052
BSCS6	0.724	0.055	0.248	0.004	–0.166	0.716	0.046	0.300	–0.070
BSCS13	0.470	0.380	–0.259	–0.108	–0.422	–	–	–	–
BSCS17	–0.012	0.052	0.063	0.101	0.909	–	–	–	–
BSCS22	0.018	0.784	0.307	0.014	0.044	0.013	0.788	0.293	0.008
BSCS28	0.114	0.045	0.101	0.813	0.186	0.069	0.017	0.177	0.874
BSCS29	0.253	0.203	0.235	0.724	–0.049	0.312	0.246	0.200	0.655
BSCS30	–0.083	0.726	0.262	0.220	–0.163	–0.026	0.768	0.201	0.129
BSCS31	0.671	0.128	0.027	0.322	0.198	0.693	0.137	0.026	0.342
BSCS32	0.675	0.070	0.117	0.300	–0.086	0.762	0.122	0.060	0.199

### Construct Validity

Table 5 shows the results of the CFA of the BSCS. Model 1 evaluated all of the 13-items of BSCS based on a single factor. The results indicated that the scale did not fit the model well, with χ^2^ (1362.277) = 65, *p* < 0.001, SRMR = 0.106, CFI = 0.873, TLI = 0.847, and RMSEA = 0.149. The five-factor model suggested in the original scale (Tangney et al., 2004) failed to obtain any results, as the fifth factor only consisted of one item, and hence the model was not identified. Model 2, which was based on the suggestions of Ferrari et al. (2009), reconceptualized the BSCS into a two-factor structure, which included general self-discipline (BSCS2, BSCS3, BSCS4, BSCS6, BSCS13, BSCS17, BSCS29, and BSCS30) and impulse control (BSCS1, BSCS28, BSCS31, and BSCS32). The CFA results also reported a poor model fit, with χ^2^ (1356.189) = 64, *p* < 0.001, SRMR = 0.106, CFI = 0.873, TLI = 0.845, and RMSEA = 0.150. Likewise, the results in Model 3 also demonstrated the other 10-item, two-factor structure of the BSCS proposed by de Ridder et al. (2011), namely, inhibition (BSCS1, BSCS2, BSCS6, BSCS17, BSCS29, and BSCS31) and initiation (BSCS3, BSCS22, BSCS28, and BSCS30). However, it failed to fulfill the cut-off criteria for a good model fit, as χ^2^ (638.066) = 34, *p* < 0.001, SRMR = 0.093, CFI = 0.904, TLI = 0.873, and RMSEA = 0.140. Model 4 evaluated a recent study that suggested an 8-item BSCS with a two-factor structure, namely, restraint (BSCS1, BSCS2, BSCS17, and BSCS22) and impulsivity (BSCS6, BSCS28, BSCS31, and BSCS32) derived from samples used in the Midwestern United States (Maloney et al., 2012). The results indicated that the two-factor structure also failed to fulfill the criteria for goodness of fit, with χ^2^ (346.287) = 19, *p* < 0.001, SRMR = 0.092, CFI = 0.886, TLI = 0.831, and RMSEA = 0.138.

**TABLE 5:** Confirmatory factor analysis of the BSCS.

**Model**	**No. of factors**	**χ^2^**	**df**	**RMSEA**	**CFI**	**TLI**	**SRMR**
**BSCS (13 items)**							
1	1	1362.277***	65	0.149	0.873	0.847	0.106
2	2	1356.189***	64	0.150	0.873	0.845	0.106
**BSCS (10 items)**							
3	2	638.066***	34	0.140	0.904	0.873	0.093
**BSCS (8 items)**							
4	2	346.287***	19	0.138	0.886	0.831	0.092
**BSCS (11 items)**							
5	4	125.391***	38	0.050	0.991	0.986	0.039
6^a^	4	106.626***	37	0.046	0.992	0.989	0.036

^*a*^Includes the covariance between the error terms for items BSCS4 and BSCS31. ***p < 0.001.

**FIGURE 1:**
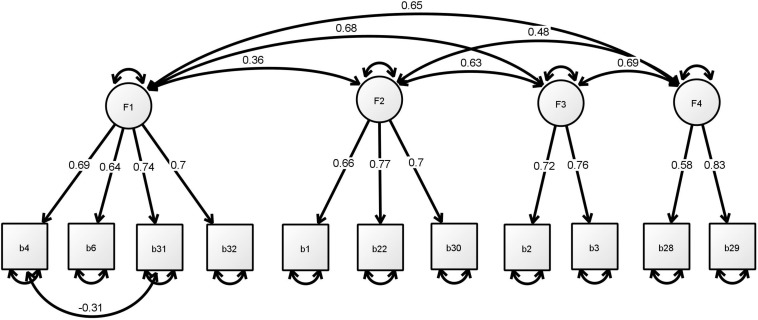
Final standardized model of the 11-item BSCS. F1, self-discipline; F2, impulsivity; F3, healthy habits; F4, self-regulation.

We propose a shortened version of the BSCS by removing two items, namely, BSCS13, factor 1 related to general capacity for self-discipline, and BSCS17, factor 5 related to reliability, based on the findings of prior analyses. The 11-item BSCS consisted of a four-factor structure, namely, F1) self-discipline: BSCS4, BSCS6, BSCS31, and BSCS32; F2) impulsivity: BSCS1, BSCS22, and BSCS30; F3) healthy habits: BSCS2 and BSCS3; and F4) self-regulation: BSCS28 and BSCS29. The CFA in Model 5 was conducted without correlating the error terms and the results were very close to the criteria of a goodness of fit other than χ^2^/df value = 3.30. Model 6 re-evaluated the 11-item BSCS, with the error correlations based on the modification indices, and it included one covariance factor between the error terms for BSCS4 and BSCS31. The data suggest that the shortened version is suitable for a four-factor scale with *post hoc* modification. The results indicated good model fit, as χ^2^ (106.626)/37 = 2.88, SRMR = 0.036, CFI = 0.992, TLI = 0.989, RMSEA = 0.046. In addition, the omega total (ω*t*) recorded 0.86, which indicated above the acceptable range. Figure 1 presents the final standardized model 1. In short, the results suggest that the 11-item BSCS comprised of items 1, 2, 3, 4, 6, 22, 28, 29, 30, 31, and 32 with a four-factor structure is an appropriate measure of self-control amongst the Chinese university student population.

## Discussion

The main contribution of this study is the re-examination of the psychometric properties and dimensionality of the BSCS in mainland China. The findings of this study suggest that a shortened version of the 11-item BSCS with a four-factor structure had better psychometric properties and good model fit in the CFA of Chinese college students. The revised version removed BSCS13 and BSCS17, and included the following four factors: self-discipline (BSCS4, BSCS6, BSCS31, and BSCS32), impulsivity (BSCS1, BSCS22, and BSCS30), healthy habits (BSCS2 and BSCS3) and self-regulation (BSCS28 and BSCS29). In terms of psychometric properties, the revised Chinese translated version of the 11-item BSCS had a high degree of internal consistency with a Cronbach’s alpha of 0.81. Both the 11-item BSCS and the 13-item BSCS demonstrated very strong and significant positive correlations with *r* = 0.988, *p* < 0.001. The revised scale also had good criterion validity with other well-established scales that are theoretically and conceptually related to self-control. The 11-item BSCS displayed good criterion validity with other construct-related scales and showed a significant moderate relation with self-esteem (RSES, *r* = 0.469), quality of life (SWLS, *r* = 0.305; WHO-5, *r* = 0.246), happiness (SHS, *r* = 0.337), and minor psychological disorders (GHQ-12, *r* = −0.428).

With regard to the controversy related to the dimensionality of BSCS, we had examined the five-factor (Tangney et al., 2004; Unger et al., 2016), two-factor (Ferrari et al., 2009; de Ridder et al., 2011; Maloney et al., 2012) and single factor constructs (Lindner et al., 2015) using CFA. The five-factor constructs of the BSCS suggested in the original scale failed to yield CFA results as the fifth factor was potentially problematic as it consisted of only one item. The findings show that the single and two-factor constructs presented in Models 1, 2, 3, and 4 failed to achieve the adequate model fit criteria. The four-factor constructs without correlating the error terms in Model 5 with RMSEA, CFI, TLI, and SRMR values were a good fit model, but χ^2^ was significant (*p* < 0.001) probably due to the effects of the large sample size (Bentler and Bonett, 1980; Kline, 2005); hence, after the covariance in the error terms based on modification indices (Shah and Goldstein, 2006; Cole et al., 2008), Model 6 was good model fit for the constructs of the BSCS (Appendix). In short, the proposed scale in this study in general retained the original factors proposed by the original scale developers (Tangney et al., 2004). It avoided the problem of artificially rearrange the factor structure without based on any theoretical justifications.

There are several potential limitations associated with this study. First, only limited number of self-control-related scales to verify the criterion validity of the BSCS in this study. Tangney et al. (2004) used measures such as the Marlowe–Crowne Social Desirability scale, the Eating Disorder Inventory, the Michigan Alcohol Screening Test, and the Symptom Checklist 90 to evaluate the BSCS. Owing to the availability of reliable Chinese translated scales and the length of the questionnaire, we adopted other well established construct-related scales, such as the RSES, SWLS, SHS, WHO-5, and GHQ12 that are commonly used or discussed in BSCS validation studies and the literature on self-control (Rothbaum et al., 1982; Tangney, 1991, 1995; Baumeister, 1994; Tangney et al., 1996, 2004; Eisenberg et al., 1998; Fabes et al., 1999; Unger et al., 2016). The findings of this study consistently demonstrate that the BSCS possesses good criterion validity in terms of magnitude and direction with other self-control related scales suggested in the literature.

Second, the sample used in this study may also limit the generalizability of the findings given that the respondents were recruited from one Chinese university with large proportion of female population. However, this limitation may have been compensated by a relatively large sample size in the university setting with reference to the other BSCS related studies. As such, Tangney et al. (2004) managed to recruit only 351 and 255 students in their studies to develop the BSCS. More importantly, we have computed additional confirmative factor analysis on both male and female participants with the 11-item BSCS. The analysis indicated the same results as we presented in Model 6, as male students with χ^2^ (37.845)/37 = 1.02, SRMR = 0.058, CFI = 0.999, TLI = 0.999, and RMSEA = 0.014 (*n* = 111), while female students with χ^2^ (111.366)/37 = 3.0, SRMR = 0.039, CFI = 0.991, TLI = 0.987, and RMSEA = 0.050 (*n* = 792). Both results fulfilled all the cut-off criteria for good model fit.

## Future Research

To evaluate the construct validity of the scale, further studies should examine and verify the four dimensional 11-item BSCS in other Chinese populations and focus on further confirming BSCS’s validity with regard to the general public and other populations. Future studies need to make use of other population samples to establish the BSCS’s wider applicability in the future. Besides, schools, reformative agencies, and practitioners could use the BSCS along with intervention programmes to evaluate its effectiveness in strengthening participants’ self-control in the Chinese context. Finally, the concept of self-control is essential in the social and psychological context. It is conceptually related to many theories and applications, such as criminology, positive psychology, subjective well-being, and quality of life. Further exploration may provide further insights into accurately describing human behavior.

## Conclusion

To conclude, the findings show that the BSCS is reliable in Chinese culture and is applicable to Chinese college populations. The results suggested that an 11-item BSCS (without BSCS13 and BSCS17) with a four-factor structure fulfilled all the cut-off criteria for good model fit in CFA. A validated Chinese version of the BSCS provides a comprehensive and handy measure for broader research in the context of mainland China or the Chinese diaspora.

## Data Availability Statement

The dataset used and/or generated for this study is available from the corresponding author on reasonable request.

## Ethics Statement

This study was carried out in accordance with the recommendations of Statistics Law of the People’s Republic of China. All subjects gave written informed consent in accordance with the Declaration of Helsinki. The protocol was approved by the Ethical Committee of the Huashang College, Guangdong University of Business Studies.

## Author Contributions

SF: study design, data collection, data analysis, data interpretation, and manuscript preparation. CK: study design and manuscript preparation. QH: study design and data collection.

## Conflict of Interest

The authors declare that the research was conducted in the absence of any commercial or financial relationships that could be construed as a potential conflict of interest.

## Appendix

**TABLE A1:** Conceptualization of the dimensionality of the Brief Self-Control Scale.

		**13-item BSCS^#^**					**13-item BSCS**		**8-item BSCS**		**10-item BSCS**		**11-item BSCS^∧^**			
						
		**T1**	**T2**	**T3**	**T4**	**T5**	**General self-discipline**	**Impulse control**	**Impulsivity**	**Restrain**	**Inhibition**	**Initiation**	**F1**	**F2**	**F3**	**F4**
BSCS1	I am good at resisting temptation		*					*		*	*			*		
BSCS2	I have a hard time breaking bad habits (reverse scored)			*			*			*	*				*	
BSCS3	I am lazy (reverse scored)			*			*		–	–		*			*	
BSCS4	I say inappropriate things (reverse scored)	*					*		–	–	–	–	*			
BSCS6	I do certain things that are bad for me, if they are fun (reverse scored)	*					*		*		*		*			
BSCS13	I refuse things that are bad for me	*					*			*	–	–	–	–	–	–
BSCS17	I wish I had more self-discipline (reverse scored)					*	*		*		*		–	–	–	–
BSCS22	people would say that I have iron self-discipline		*				*		–	–		*		*		
BSCS28	pleasure and fun sometimes keep me from getting work done (reverse scored)				*			*	–	–		*				*
BSCS29	I have trouble concentrating				*		*		*		*					*
BSCS30	I am able to work effectively toward long-term goals		*				*		*			*		*		
BSCS31	sometimes I can’t stop myself from doing something, even if I know it is wrong (reverse scored)	*						*	–	–	*		*			
BSCS32	I often act without thinking through all the alternatives (reverse scored)	*						*		*	–	–	*			

*Source: Ferrari et al. (2009), de Ridder et al. (2011), Maloney et al. (2012), Tangney et al. (2004). ^#^T1, general capacity for self-discipline; T2, inclination toward deliberate/non-impulsive action; T3, healthy habits; T4, self-regulation in service for a work ethic; T5, reliability. ^∧^F1, self-discipline; F2, impulsivity; F3, healthy habits; F4, self-regulation.*
